# Ultralow threshold surface emitting ultraviolet lasers with semiconductor nanowires

**DOI:** 10.1038/s41598-023-33457-9

**Published:** 2023-04-24

**Authors:** Mohammad Fazel Vafadar, Songrui Zhao

**Affiliations:** Department of Electrical and Computer Engineering, McGill University, 3480 University Street, Montreal, QC H3A 0E9 Canada

**Keywords:** Nanowires, Photonic crystals

## Abstract

Surface-emitting (SE) semiconductor lasers have changed our everyday life in various ways such as communication and sensing. Expanding the operation wavelength of SE semiconductor lasers to shorter ultraviolet (UV) wavelength range further broadens the applications to disinfection, medical diagnostics, phototherapy, and so on. Nonetheless, realizing SE lasers in the UV range has remained to be a challenge. Despite of the recent breakthrough in UV SE lasers with aluminum gallium nitride (AlGaN), the electrically injected AlGaN nanowire UV lasers are based on random optical cavities, whereas AlGaN UV vertical-cavity SE lasers (VCSELs) are all through optical pumping and are all with large lasing threshold power densities in the range of several hundred kW/cm^2^ to MW/cm^2^. Herein, we report ultralow threshold, SE lasing in the UV spectral range with GaN-based epitaxial nanowire photonic crystals. Lasing at 367 nm is measured, with a threshold of only around 7 kW/cm^2^ (~ 49 μJ/cm^2^), a factor of 100× reduction compared to the previously reported conventional AlGaN UV VCSELs at similar lasing wavelengths. This is also the first achievement of nanowire photonic crystal SE lasers in the UV range. Further given the excellent electrical doping that has already been established in III-nitride nanowires, this work offers a viable path for the development of the long-sought-after semiconductor UV SE lasers.

## Introduction

SE semiconductor lasers are important for a variety of fields such as photonics, information and communication technologies, and biomedical sciences^1–6^. Compared to edge-emitting lasers, SE lasers offer a number of advantages such as low beam divergence, circular far-field pattern, fast modulation speed, two-dimensional integration capability, and so on^5, 7^. Over decades of development, gallium arsenide (GaAs)-based near-infrared (IR) SE lasers have turned into a billion-dollar industry, impacting both data communication and 3D sensing such as face recognition and time-of-flight imaging^8–12^. The success of SE lasers in the near-IR unfortunately is not seen in the shorter visible and UV spectral ranges. For example, despite of the encouraging progress in GaN-based blue and green SE lasers in recent years, they have not yet reached the same level of maturity as that of their counterparts in the near-IR^4, 10, 13–23^. In the UV range, the situation is even more lagging behind. None of the existing technologies can meet the practical application needs. Breakthrough in the UV SE laser development is pivotal to a variety of applications related to our everyday life including disinfection, medical diagnostics, phototherapy, curing, and high-resolution 3D printing^24, 25^.

At present, while there are many existing efforts in developing UV SE lasers with other material systems such as organic semiconductors and zinc oxide (ZnO), as well as other photonic technologies such as coupling nonlinear optics to near-IR GaAs-based VCSELs, e.g., Refs.^26–31^. AlGaN has received a wide interest for the UV SE laser development due to a number of advantages such as direct, ultrawide, and tunable bandgap energies, chemically stable, mechanically strong, highly compact, and so on. Nonetheless, the electrically injected AlGaN nanowire UV SE lasers demonstrated hitherto are all based on random optical cavities^32–35^, whereas AlGaN UV VCSELs are all through optical pumping and are all with large lasing threshold power densities^8, 11, 36–45^. For instance, the threshold power density for sub-280 nm lasing is 1.2 MW/cm^2^^39^, and even for lasing at longer wavelength (e.g., close to 400 nm) the threshold power density is in the range of around 200–400 kW/cm^2^^11, 40^. Herein, we demonstrate ultralow threshold, SE lasing in the UV spectral range using GaN-based epitaxial nanowire photonic crystal (epi-NPC) structures, which cannot only overcome the drawbacks of random optical cavities with self-organized nanowires, but also greatly mitigate the challenges in conventional AlGaN UV VCSELs. The UV SE lasing shown in this study is at 367 nm with a threshold of merely 7 kW/cm^2^, a 100× reduction compared to the conventional AlGaN UV VCSELs. The use of photonic crystal-based SE lasers can also potentially offer uniform single mode over a large area and other benefits such as on-demand beam^12^.

**Figure 1:**
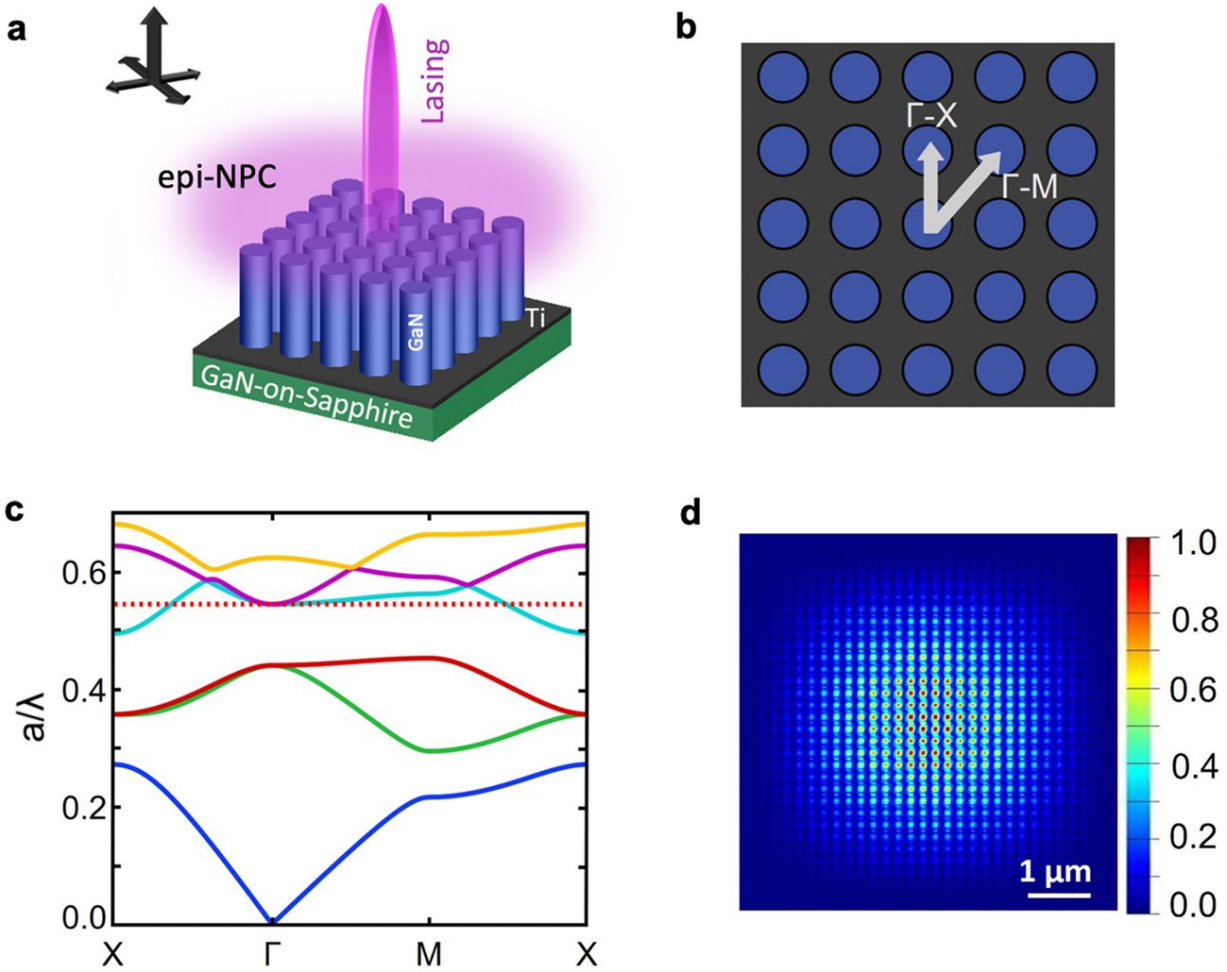
(**a**) Schematic of the UV SE lasing using GaN epi-NPC. Inset: In-plane light propagation and diffraction normal to the plane. (**b**) Top view of the NPC structure, with two specific directions Γ-X and Γ-M labeled. (**c**) Photonic bands of the NPC structure with *a* = 200 nm and *d*_NW_ = 173 nm. The red dot line indicates the reduced frequency corresponds to *λ* ~ 367 nm. (**d**) The electric field profile (|*E*|^2^) of the band edge mode calculated by the 3D FDTD method.

A schematic illustration of the device concept is shown in Fig. 1a, which utilizes GaN epi-NPC arranged in a square lattice for the optical cavity formation to achieve SE lasing. The use of square lattice is favorable for single mode lasing as well as realizing various functionalities, e.g., Refs.^12, 46^. An illustration of the in-plane light beam propagation and the diffraction to the normal direction forming SE lasing is also shown in the inset of Fig. 1a. Figure 1b shows the top view of such NPC, with two specific directions Γ-X and Γ-M labeled. For GaN, the band-edge light emission is around 364 nm^47^. Therefore, we design an NPC structure that can form a cavity to support lasing around this wavelength. Figure 1c shows the 2-dimensional (2D) transverse-magnetic (TM) photonic band structure using 2D space and wave optics package in COMSOL Multiphysics, with a lattice constant (*a*, center to center distance) of 200 nm and a nanowire diameter (*d*_NW_) of 173 nm. The dot line represents the reduced frequency (*a/λ*). In general, at photonic band edges, the light group velocity becomes zero, i.e., *dω/dk* → 0, so that standing waves can be formed, and lasing can be achieved using such slow light, due to a significantly enhanced interaction time between the radiation field and gain medium^19, 21, 22^. From Fig. 1c, it is seen that the reduced frequency aligns to band edges at Γ point with *a/λ* ~ 0.545, suggesting the formation of a standing wave and a possible lasing (if gain is greater than loss) at this point, with *λ* ~ 367 nm. Moreover, at Γ point, the light beam can also be diffracted normal to the photonic crystal plane, forming SE lasing^12, 46, 48–50^. Figure 1d further shows the mode profile (|*E*|^2^) of the designed NPC structure, simulated using three-dimensional (3D) finite difference time-domain (FDTD) method. It is seen that strong mode intensity is observed in the NPC. In the FDTD simulation, the nanowires with the same design parameters as mentioned above were arranged in a square lattice on a GaN substrate. A TM dipole source with a central wavelength of 367 nm was positioned in the center of the nanowire array. The lateral dimension for the simulation was 6 μm × 6 μm, and the perfectly matched layer (PML) boundary condition was used.

Experimentally, the NPC structure was formed on a patterned GaN-on-sapphire substrate using molecular beam epitaxy (MBE). To form the pattern, 10 nm Ti was first deposited using an electron beam evaporator, which was followed by electron beam lithography (EBL) and reactive ion etching (RIE) to create nanoholes with different diameters (*a* = 200 nm) arranged in a square lattice. To form the NPC, it followed a two-step process. Ti-patterned substrate was first nitrided at 400 °C in the MBE growth chamber, to prevent crack and degradation at elevated temperatures. This was followed by the growth of GaN nanowires. The growth condition included a substrate temperature (*T*_sub_) of 865 °C, a nitrogen flow rate of 0.9 sccm, and a Ga flux of 2.5 × 10^−7^ Torr. Detailed growth condition analysis can be found elsewhere^51^.

**Figure 2:**
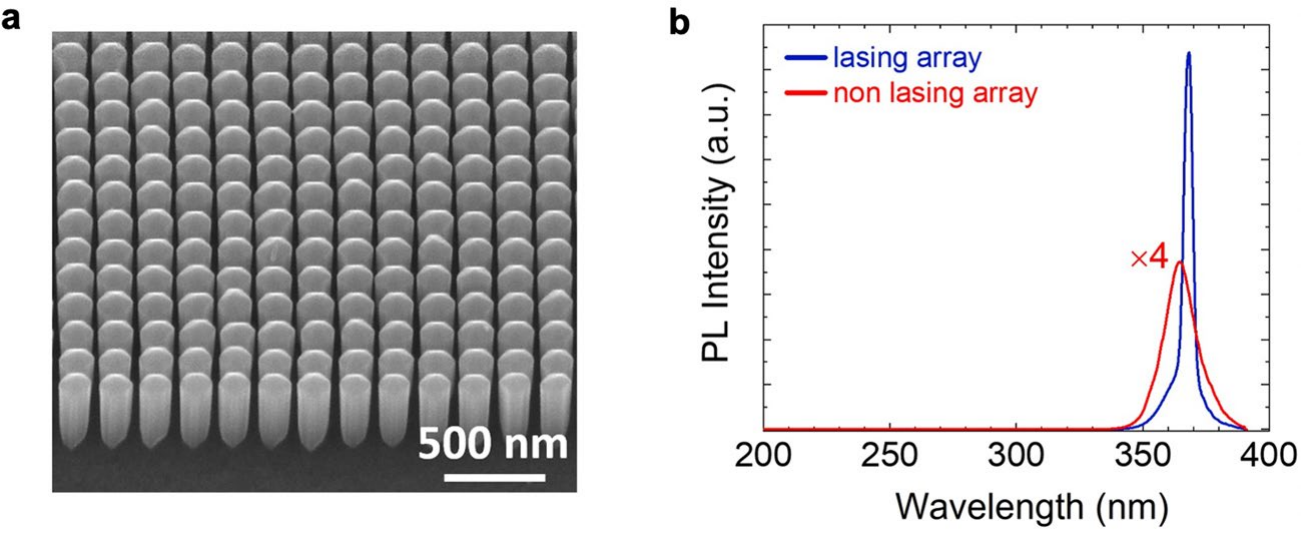
(**a**) A tilted-view SEM image of the NPC structure (the lasing array). (**b**) The RTPL spectra of the lasing and non-lasing arrays. The SEM image of the non-lasing array can be found in Supp. Info.

The dimension for the grown NPC was 75 μm × 75 μm, with edges parallel to the edges of the wafer that had a size of 1 cm × 1 cm. An optical image of the array is shown in Fig. S1a. A scanning electron microscope (SEM) image of the NPC is shown in Fig. 2a. The SEM image was taken at a tilting angle of 45° using a field-emission (FE) SEM. It is seen that the nanowires are highly uniform. Detailed examination further confirms that the nanowires have a similar uniformity at a large scale. The large-scale SEM images are shown in Fig. S1b–d. Statistics on the nanowire diameter was further carried out using SEM images, which gives an average *d*_NW_ of 173.2 nm and a standard deviation of 4.4 nm (This error bar might be largely limited by the EBL process). As such, a large-area NPC that is close to the design (with respect to the nanowire diameter) is obtained experimentally.

Figure 2b shows the room-temperature (RT) photoluminescence (PL) spectrum collected from the top surface of the NPC structure (denoted as “lasing array”), excited by a 213 nm pulse laser (pulse width: 7 ns; repetition rate: 200 Hz) under a peak power density of 63.5 kW/cm^2^. The laser light was focused onto the sample surface through a focus lens (spot size: ~ 9 × 10^–4^ cm^2^), and the emitted light was also collected from the sample surface using a focus lens (NA ~ 0.31), which was further coupled to an optical fiber and an UV spectrometer (QE Pro, spectral resolution ~ 0.3 nm). Also shown in Fig. 2b is the PL spectrum of an array of *a* = 600 nm and *d*_NW_ = 325 nm (denoted as “non-lasing array”) measured under the same condition. The SEM image of the non-lasing array is shown in Fig. S2a. The photonic band structure of the non-lasing array was also calculated and is shown in Fig. S2b. It is found that the reduced frequency *a*/*λ* (*λ* = 367 nm) does not correlate to any band edge modes, suggesting the absence of light amplification. This is consistent with what is shown in Fig. 2b: While a strong PL emission is measured from the lasing array with a narrow linewidth, the PL emission from the non-lasing array is much weaker (roughly reduced by a factor of 10) with the linewidth remaining broad (a full-width half-maximum of ~ 15 nm). Moreover, the PL peak position of the non-lasing array is at around 364 nm, consistent with the band-edge emission of GaN; whereas for the lasing array, the PL peak is shifted to a longer wavelength, due to the optical cavity.

**Figure 3:**
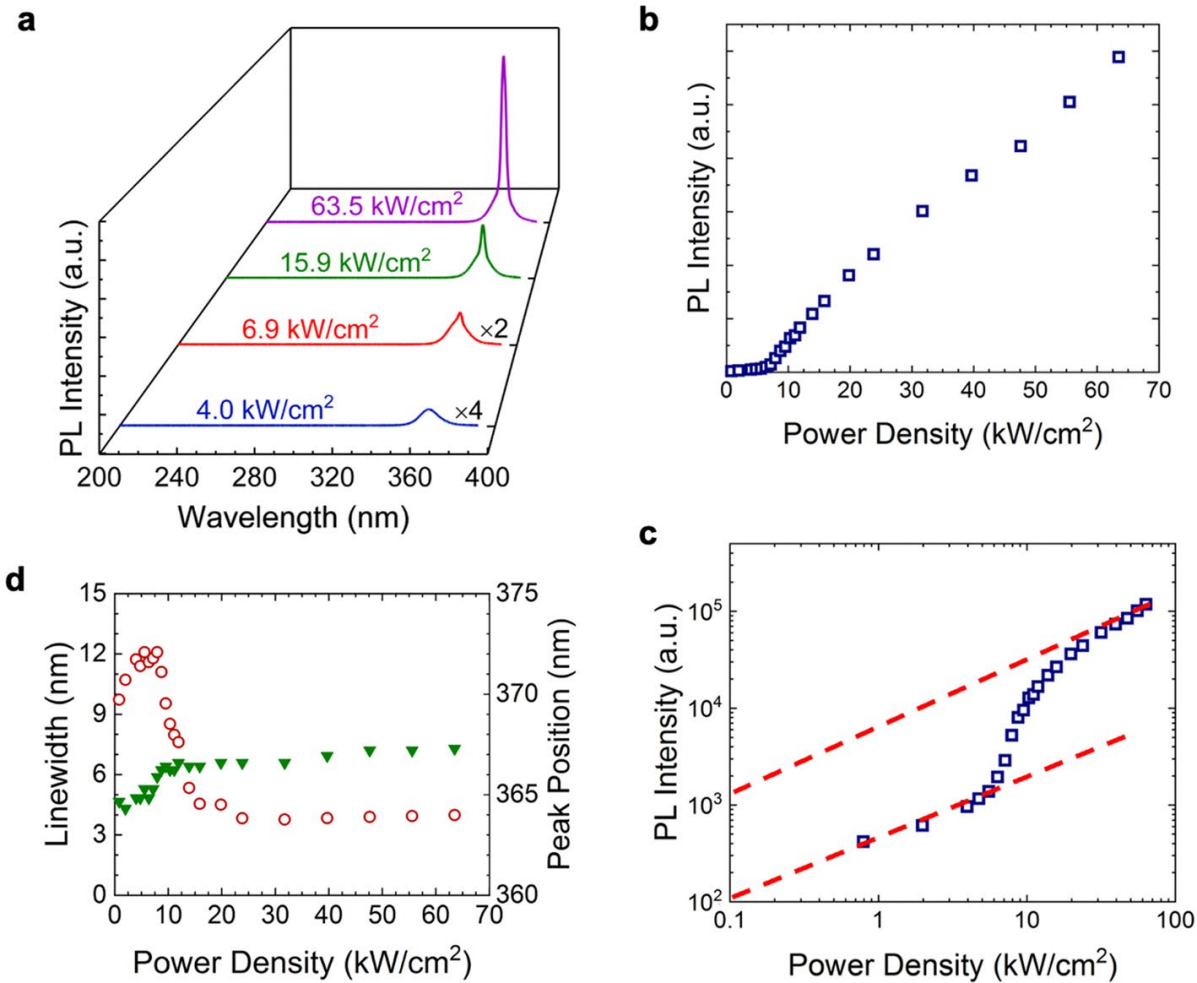
(Clockwise) (**a**) The light emission spectra of the NPC structure under different excitation powers. The light intensity versus the peak power density in a linear scale (**b**) and in a logarithmic scale (**c**). (**d**) Linewidth (open symbols) and the light emission peak position (filled symbols) versus the peak power density. Dash lines are a guide for eyes.

Detailed measurements further confirm the achievement of an ultralow threshold SE lasing. Shown in Fig. 3a are the light emission spectra under different excitation densities. It is seen that as the excitation density increases, the spectra become narrow, accompanied by a rapid increase of the light intensity. This trend is more clearly shown by the L–L (light-out versus light-in) curve in Fig. 3b, with a clear threshold around 7 kW/cm^2^. The lasing is further confirmed by examining the L–L curve in a logarithmic scale. As shown in Fig. 3c, a clear S-shape, corresponding to the spontaneous emission (linear), the amplified spontaneous emission (super linear), and the lasing (linear), is observed, being the confirmative evidence for lasing^32–34^.

It is further noted that, in this study, the lasing light intensity collected from the side is only ~ 1/30 compared to that collected from the top, suggesting the surface dominated light emission. Detailed discussions can be found in Supp. Info. Text S3. In this study, we have also measured the PL spectra of GaN-on-sapphire template and GaN-on-sapphire with Ti mask. The results are described in Supp. Info. Text S4. Briefly, only weak PL is measured from GaN-on-sapphire with Ti mask, suggesting that the light emission measured from both the non-lasing and lasing arrays are from the GaN nanowires grown on top. This also confirms that the lasing is due to the light emission from the NPC. It is also noted that, as the lasing array and the non-lasing array have the same height, it rules out that the lasing is due to the formation of a Fabry–Perot (FP) cavity.

The spontaneous emission coupling factor *β* was further estimated by using the intensity ratio of the spontaneous emission versus the lasing emission, as indicated by the dashed lines in Fig. 3c. A *β* factor of around 0.08 can be derived. This *β* factor is comparable to the previously reported photonic crystal SE lasers and is larger compared to the values reported in conventional AlGaN UV VCSELs, due to the efficient photon coupling in a photonic crystal cavity^8, 11, 14, 23^. Figure 3d shows the linewidth and the peak wavelength as a function of the excitation power. A clear reduction of the linewidth near the threshold is seen. The relatively broad linewidth could be related to multiple lasing modes. Moreover, it is also seen that after the threshold the peak wavelength is nearly unchanged, suggesting a nearly stable lasing wavelength.

**Figure 4:**
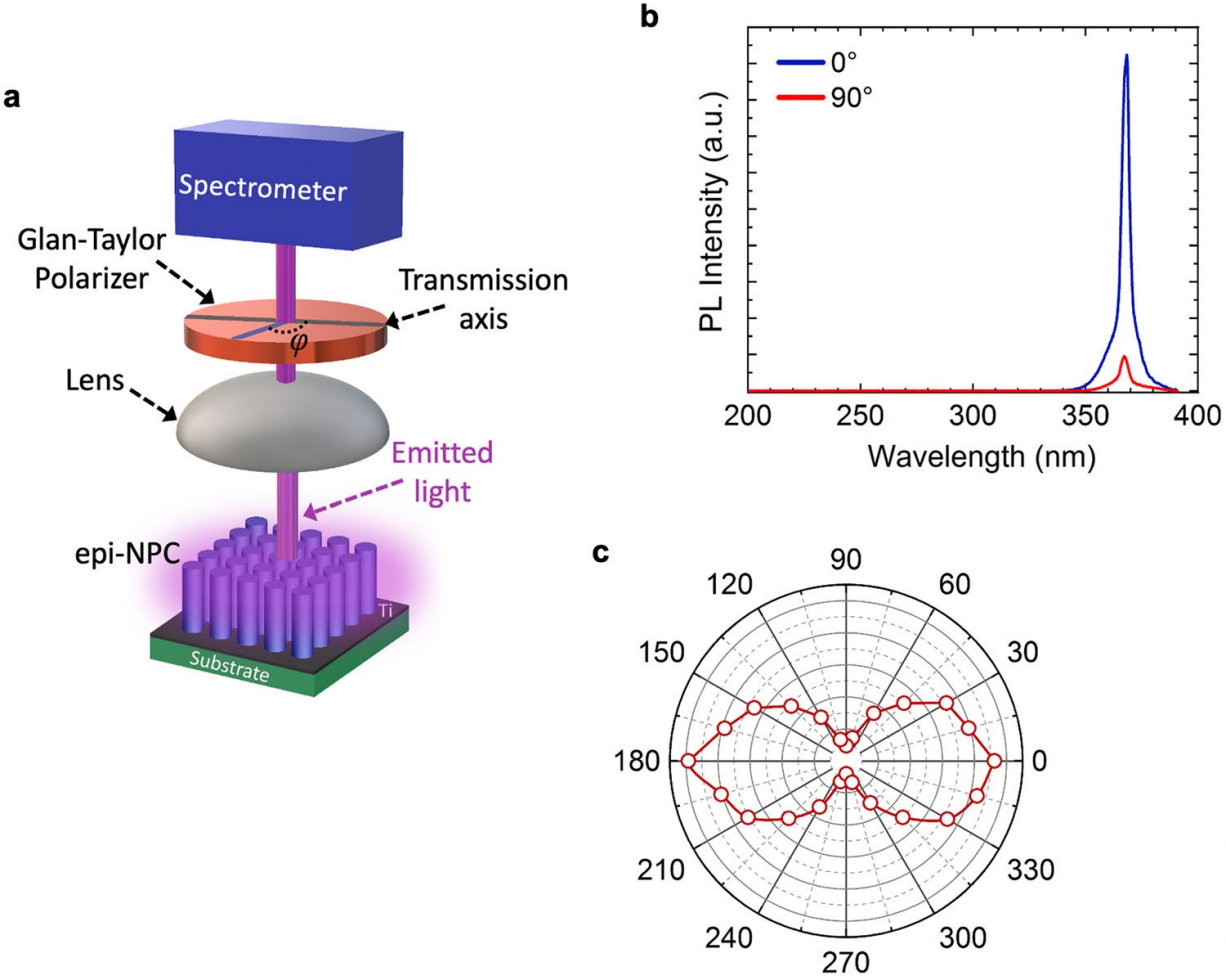
(**a**) The schematic of the in-plane polarization measurement at Γ point. (**b**) Polarized light emission from the NPC at *φ* = 0° and *φ* = 90°. (**c**) Plot of the light intensity measured from the NPC at different in-plane angle *φ*. The excitation density was 63.5 kW/cm^2^.

The in-plane polarization at Γ point is investigated in the end. In this regard, the light emission was collected from the device top with a polarizer inserted in the light collection path, whereas the pumping end is similar to that described earlier for the results shown in Figs. 2 and 3. The collection end is schematically shown in Fig. 4a: a Glan–Taylor polarizer is placed in the light collection path, and the in-plane angle *φ* is also labeled. Here, *φ* = 0° means the electric field is along the transmission axis of the polarizer. From Fig. 4b, it is seen that the light intensity at *φ* = 0° is roughly about 10 times stronger compared to the light intensity at *φ* = 90°, suggesting the emitted light is highly polarized in-plane at Γ point. Figure 4c further shows the light intensity at various angle *φ*. If defining the polarization ratio (degree of polarization) *ρ* = (*I*_max_* − I*_min_)/(*I*_max_ + *I*_min_), a *ρ* value of around 0.8 is obtained, suggesting a high degree of in-plane polarization. Similar polarization behavior has been reported previously from InGaN-based photonic crystal SE lasers^14, 19, 21, 23^. The in-plane polarization behavior in the present study could be related to multiple lasing modes, and the detailed mechanism is being investigated.

**Figure 5:**
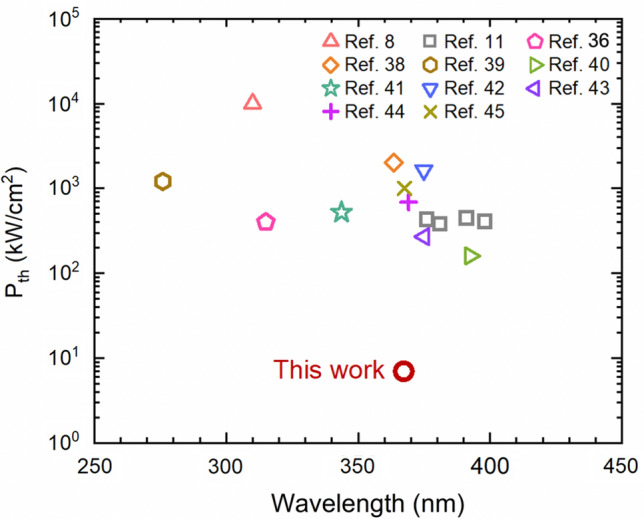
Comparison of the lasing threshold power density (P_th_): the previously reported conventional AlGaN UV VCSELs versus the NPC UV SE lasers in this study. Dash line is a guide for eyes.

Figure 5 shows the comparison plot of the lasing threshold achieved in this study versus the lasing thresholds from the previously reported conventional AlGaN UV VCSELs at different wavelengths. It is seen that, for the conventional AlGaN UV VCSELs, the lasing threshold is in the range of several hundred kW/cm^2^ to MW/cm^2^, and the lasing threshold increases as the lasing wavelength becomes shorter, as indicated by the dash line. For lasing at wavelengths similar to the wavelength in the present study, the threshold is around 0.7–1 MW/cm^2^. In contrast, the lasing threshold in the present study is only around 7 kW/cm^2^.

For the conventional AlGaN UV VCSELs, the primary challenges lie in the difficulty in obtaining high-quality distributed Bragg reflector (DBR) mirrors (primarily limited by the material quality due to large lattice mismatches), the difficulty in obtaining low resistivity AlGaN due to the poor electrical doping (primarily p-type), and the complexity in the device fabrication process, e.g., Refs.^8, 11^. Using epitaxial nanowire photonic crystals can greatly mitigate these challenges. For example, the bottom-up nanowires have been proved to be able to improve the material quality due to the efficient strain relaxation to the large surface area, e.g., Refs.^47, 52, 53^. Further, exploiting the band-edge modes of photonic crystals for lasing can avoid the problematic DBR mirrors for the cavity formation. This largely contributes to the ultralow threshold UV SE lasing achieved in this study, compared to the conventional AlGaN UV VCSELs.

Another important reason for achieving the ultralow threshold UV SE lasing in this study is the formation of a large-scale high-quality NPC experimentally. In order to have such an NPC, a close match to the design is critical. We have previously established the correlation of the lateral growth rate to the growth condition and pattern design, using the low-temperature selective area epitaxy (LT-SAE)^51^; and in this study, extensive MBE growth and substrate patterning were further carried out, partially due to the error bar in the EBL process. In addition, the significantly improved selective area epitaxy by LT-SAE could be another factor that contributes to the large-scale high-quality NPC^51^.

In summary, in this work we have demonstrated ultralow threshold, SE lasing in the UV spectral range using GaN epi-NPC. The lasing wavelength is at 367 nm, with a threshold of merely 7 kW/cm^2^ (or ~ 49 μJ/cm^2^), two orders of magnitude lower compared to the previously reported conventional AlGaN UV VCSELs at similar lasing wavelengths. This lasing threshold is also more than one order of magnitude lower compared to the conventional AlGaN VCSELs at the near-UV spectral range. Further given the excellent electrical doping that has already been established in III-nitride nanowires^54–56^ and the fully epitaxial process, this study provides a viable path for the development of electrically injected SE semiconductor lasers in the UV range, with controlled beam properties, in contrast to the previously demonstrated electrically injected UV random lasers with semiconductor nanowires, as well as the integration capability to other existing semiconductor device platforms for increased functionalities.

## Supplementary Information

Supplementary Information.

## Data Availability

The data is available upon reasonable request to the corresponding author.
