# Supplementary material for: Ultralow threshold surface emitting ultraviolet lasers with semiconductor nanowires
Source: Sci Rep. 2023 Apr 24;13:6633. doi: 10.1038/s41598-023-33457-9 (PMC10126006; doi:10.1038/s41598-023-33457-9)
Supplement: Supplementary file 1 — Supplementary Information. [file 41598_2023_33457_MOESM1_ESM.docx]

**Supplementary Information**

**Ultralow Threshold Surface Emitting Ultraviolet Lasers with Semiconductor Nanowires**

Mohammad Fazel Vafadar and Songrui Zhao*

*Department of Electrical and Computer Engineering, McGill University, 3480 University Street, Montreal, Quebec H3A 0E9, Canada*

**Email:* [*songrui.zhao@mcgill.ca*](mailto:songrui.zhao@mcgill.ca)*. Phone: +1-514-398-3244*

**Text S1: Illustration of the uniformity of the MBE-grown NPC lasing array at a large scale**

The NPC lasing array had a square shape, with the side dimension of 75 μm. The optical image and large-scale SEM images are shown below in Figure S1.

**
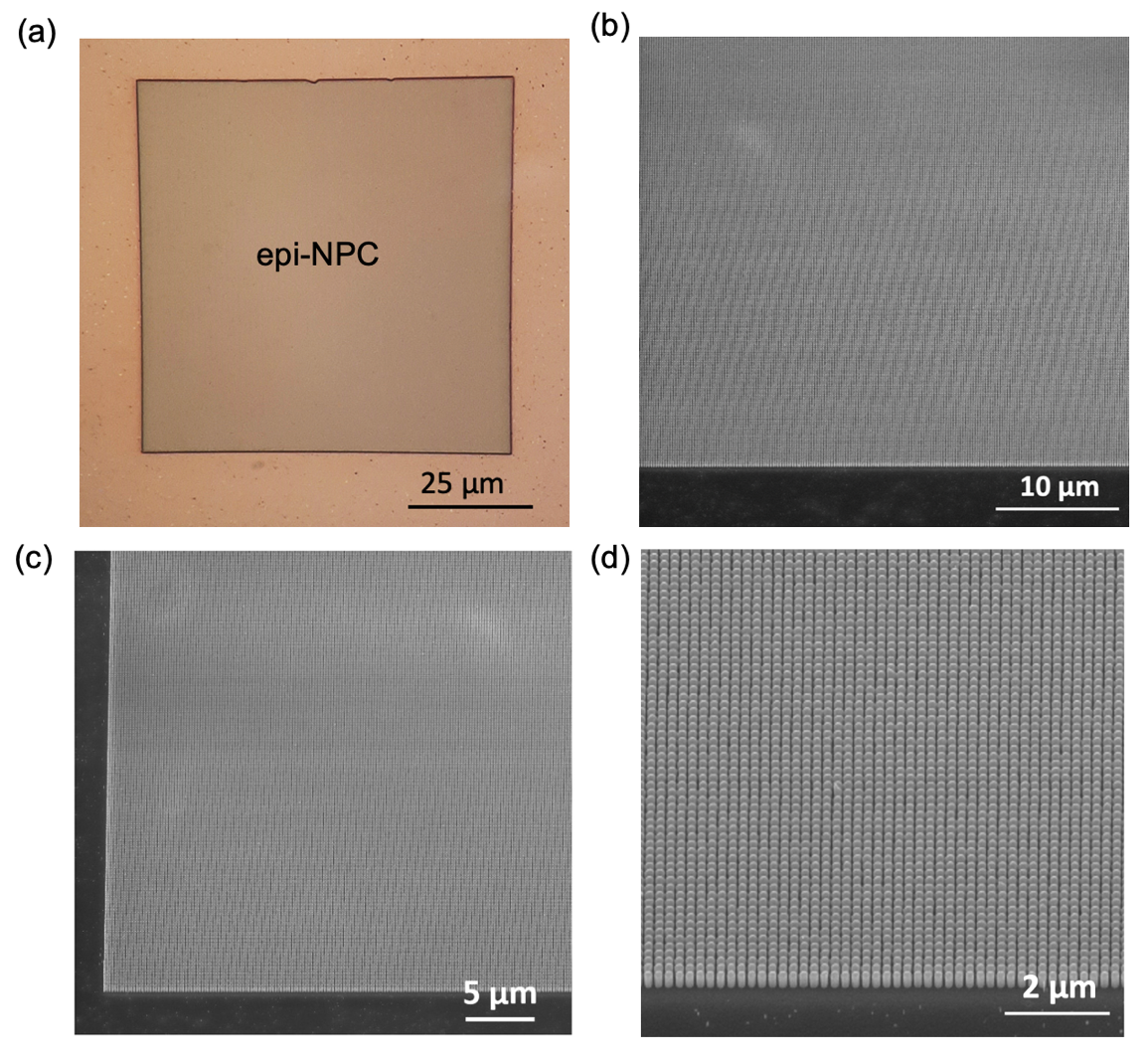
**

**Figure S1.** (a) An optical image of the array. (b) to (d) Large-scale SEM images of the NPC lasing array. The viewing angle is 45°.

**Text S2: Non-lasing array details**

Figure S2(a) shows the SEM image of the non-lasing array (*a* = 600 nm and *d*_NW_ = 325 nm). The simulated photonic band structure of the non-lasing array is shown in Figure S2(b). *λ* = 367 nm correlates to a reduced frequency (*a/λ*) of ~1.63, indicated by the dot line, which does not correlate to any band edge modes.


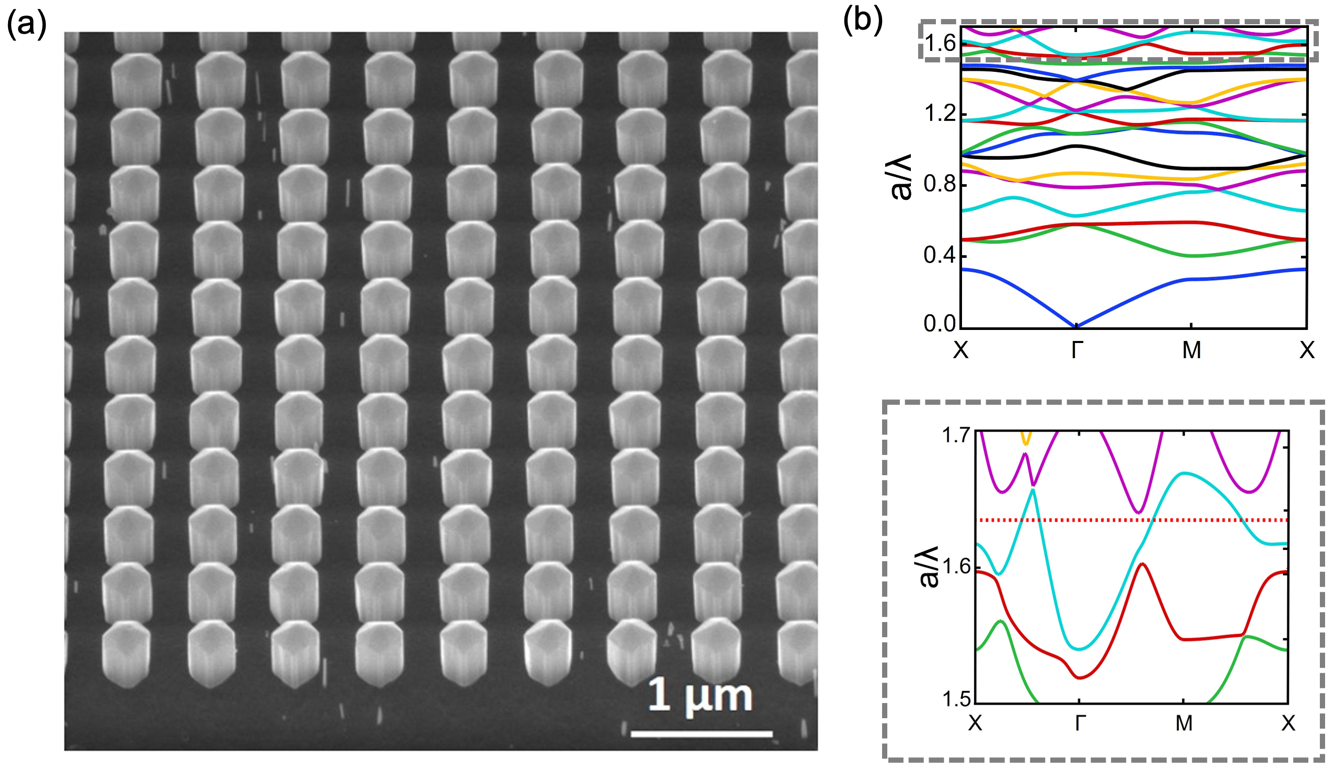


**Figure S2.** (a) SEM image of the non-lasing array. (b) Photonic band structure of the non-lasing array.

**Text S3: RTPL spectra from the top and side of the NPC lasing array**

Figure S3 shows the RTPL spectra of the lasing array measured from the top (0° with respect to the normal direction) and the side (85° with respect to the normal direction) under a peak power density of 69.7 kW/cm^2^. It is seen that the lasing light intensity collected from the top surface is roughly 30× higher compared to that collected from the side, confirming the surface dominated light emission.


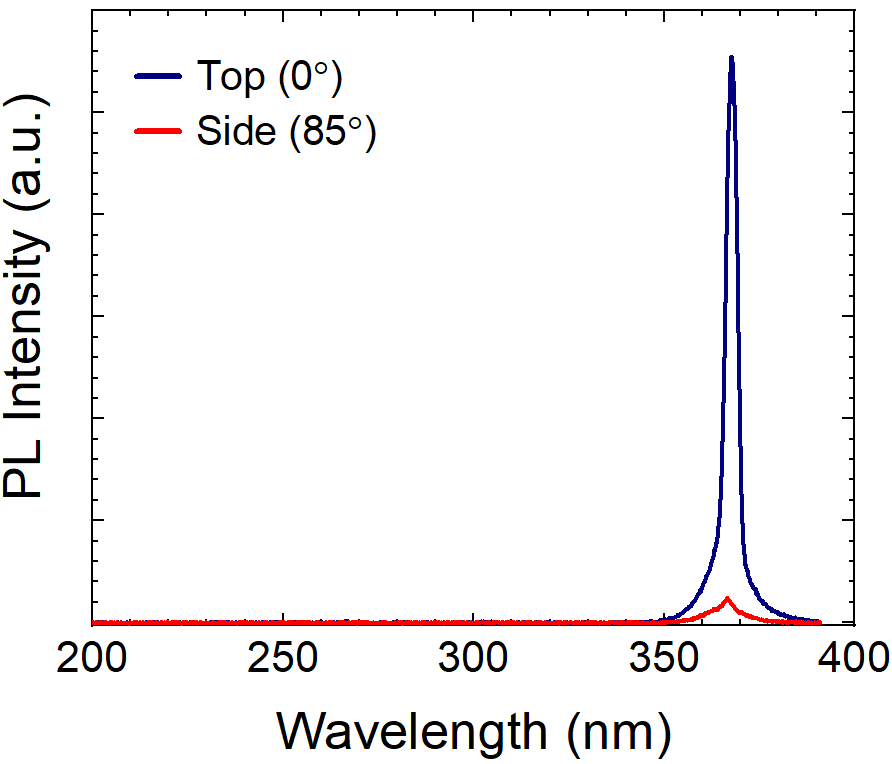


**Figure S3.** RTPL of the NPC lasing array collected from the top and the side.

**Text S4: RTPL spectra of the GaN-on-sapphire template and the GaN-on sapphire with Ti mask**

Figure S4 shows the RTPL spectra of the GaN-on-sapphire template and the GaN-on-sapphire with Ti mask under a peak power density of 63.5 kW/cm^2^, using the same setup for the measurements of the lasing and non-lasing arrays. The extremely weak PL from the GaN-on-sapphire with Ti mask confirms that the light emission measured from the lasing and non-lasing array is from GaN nanowires grown on top.


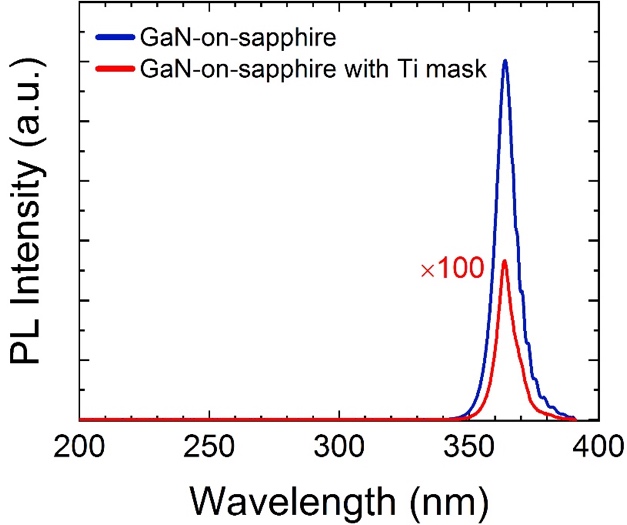


**Figure S4.** RTPL of the GaN-on-sapphire template and the GaN-on sapphire with Ti mask.
